# Association between Intimate Partner Violence during Pregnancy and Adverse Pregnancy Outcomes in Vietnam: A Prospective Cohort Study

**DOI:** 10.1371/journal.pone.0162844

**Published:** 2016-09-15

**Authors:** Thanh Nguyen Hoang, Toan Ngo Van, Tine Gammeltoft, Dan W. Meyrowitsch, Hanh Nguyen Thi Thuy, Vibeke Rasch

**Affiliations:** 1 Institute of Preventive Medicine and Public Health, Hanoi Medical University, Hanoi, Vietnam; 2 Department of Anthropology, University of Copenhagen, Copenhagen, Denmark; 3 Department of Public Health, University of Copenhagen, Copenhagen, Denmark; 4 Department of Obstetrics and Gynecology, Odense University Hospital, Odense, Denmark; 5 Department of Clinical Research, University of Southern Denmark, Odense, Denmark; Stony Brook University, Graduate Program in Public Health, UNITED STATES

## Abstract

**Background:**

Violence against pregnant women is an increasing public health concern particularly in low- and middle-income countries. The purpose of this study was to measure the association between intimate partner violence (IPV) during pregnancy and the risk of adverse birth outcomes.

**Methods:**

Prospective cohort study of 1276 pregnant women in Dong Anh district, Vietnam. Women with gestational age less than 24 weeks were enrolled and interviewed. Repeated interviews were performed at 30–34 weeks gestation to assess experience of IPV during pregnancy and again 48 hours post-delivery to assess the birth outcome including birth weight and gestational age at delivery.

**Results:**

There was a statistically significant association between exposure to physical violence during pregnancy and preterm birth (PTB) or low birth weight (LBW). After adjustment for age, education, occupation, body mass index (BMI), haemoglobin level, previous adverse pregnancy outcomes, the pregnant women who were exposed to physical violence during pregnancy were five times more likely to have PTB (AOR = 5.5; 95%CI: 2.1–14.1) and were nearly six times more likely to give birth to a child of LBW (AOR = 5.7; 95%CI: 2.2–14.9) as compared to those who were not exposed to physical violence.

**Conclusion:**

Exposure to IPV during pregnancy increases the risk of PTB and LBW. Case-finding for violence in relation to antenatal care may help protect pregnant women and improve pregnancy outcomes.

## Introduction

Intimate Partner Violence (IPV) is an increasing public health concern particularly in low- and middle-income countries [1, 2]. According to a recent report from World Health Organisation (WHO), 35% of women worldwide have experienced either physical and/or sexual violence [3]. Pregnant women constitute a particularly vulnerable sub-group, with prevalence rates of IPV during pregnancy ranging between 4% and 29% [4]. IPV during pregnancy does not only affect the women’s health, but also imposes adverse health effect of the newborn children and their later development [5–7]. Preterm birth (PTB), defined as birth of infant before 37 weeks of gestation and low birth weight (LBW), defined as birth weight <2500g [8] are leading causes of neonatal, infant, and childhood morbidity, mortality as well as disabilities [5, 8]. Studies have demonstrated an association between violence during pregnancy and LBW or PTB, however most of the studies have been performed in developing countries and have mainly relied on either cross-sectional or case-control designs and have thus not been appropriate for documenting a causal relationship between IPV and PTB or LBW. There is a need of larger prospective studies from low- and middle-income countries, which describes how different forms of IPV may be associated with PTB and LBW in such settings [9–12]. In Vietnam, 63.000 children aged less than 5 year die every year and 50% of these deaths occur among newborns. One of the main causes of newborn deaths are due to complications of prematurity and LBW, which together accounts for almost 50% of all newborn deaths [13]. The National Study on Violence against Women in Vietnam in 2010 showed that 58% of women have suffered violence at least once in their life (including physical, mental and sexual violence). Focusing on physical violence, 32% of ever-married women report to have lifetime experience of physical violence and 6% have experienced physical violence during the past 12 months. The prevalence of physical violence during pregnancy in Vietnam has been reported to be 5% [14]. Antenatal care programs in Vietnam do acknowledge that maternal health affect the health of the child, however the role of violence as an underlying factors in women’s health during pregnancy and postpartum, remains an area where robust evidence is lacking. The present study aims to help closing this knowledge gap by providing information on the occurrence of IPV among pregnant women and how it is associated with LBW and PTB in Vietnam.

## Materials and Methods

### Study deign and setting

A cohort study performed among women attending antenatal care in Dong Anh District, which is located 15 kilometre North-west of Hanoi capital of Vietnam. The women were recruited from all 24 communes in Dong Anh District between June 2014 and July 2015.

### Participants, sample size and data collection procedure

Participants were pregnant women before 24 weeks of gestation in Dong Anh district. The sample size calculated based on cohort study formula. An appropriate sample size was calculated based on the assumption that 41.2% of the women were exposed to IPV and delivered PTB infants, whereas 20.6% were not exposed to IPV and delivered PTB infants [15]. For LBW, the similar proportional distributions were 42.3% and 21.2%, respectively [15]. The results of the two analyses (considering a risk ratio = 2; precision = 0.15; power: 90%) indicated an appropriate sample size of 1044 and 1084 women, respectively. The assumed loss to follow-up was 20%. Hence, a total sample of 1300 pregnant women was needed in order to test the hypothesis regarding an association between exposure to IPV and the two adverse pregnancy outcomes.

List of all pregnant women less than 24 weeks of gestation age (from March 2014 to July 2015) in Dong Anh district was established by the collaborators of the commune's population. Based on this list the research team sent the invitation letter to all them to go to ANC at two district hospitals or commune health Centre. If they were less than 24 weeks of gestation age (determined by ultrasound), did not suffer from chronic diseases and to agree to participate in research, they were selected. Data collection was conducted in three stages: at enrolment where information on socio-demographic and reproductive health was collected; at 30–34 weeks of pregnancy where detailed information on exposure to violence before and during pregnancy was collected and at delivery where the birth weight and gestational age of the child were determined.

**Fig 1:**
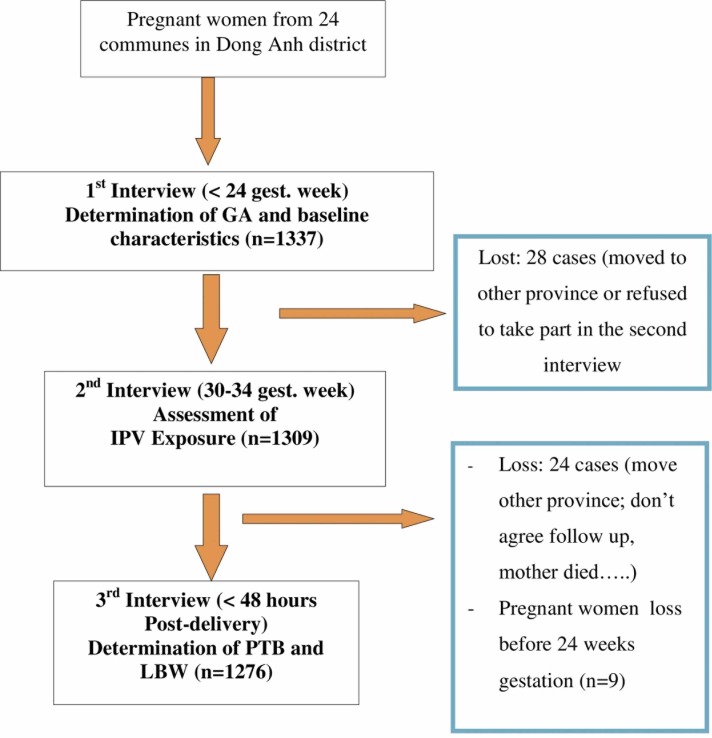
Follow-up chart.

In fact 1,337 pregnant women were enrolled before 24 weeks of gestation (confirmed by ultrasound scanning), 1276 (96%) of these women were followed from inclusion until delivery. The processing of data collection is presented in Fig 1.

### Questionnaires and interviewers

A team of six interviewers who were selected among the staff in Dong Anh Population Centre district were trained and supervised by the research team from Hanoi Medical University, University of Copenhagen, and University of Southern Denmark. The questionnaires were developed based on the WHO questionnaire on health and violence against women which has been validated and used in the National Study on Violence against Women in Vietnam in 2010 [14]. To capture violence enacted during pregnancy, the IPV assessment period of “past 12 months” in the original tool was changed to “during this pregnancy”. The adapted questionnaire was pilot tested and revised according to the gained experience.

### Measure

#### IPV

Maternal exposure to IPV was determined through the question: ‘‘during your present pregnancy did your current partner or boyfriend do any of the following things to you? The list of potential offences were as follows: *Physical violence*: slapped, pushed, hit, kicked, chocked or threatened to use or actually used anything that could hurt them; *Emotional violence*: insulted, humiliated, intimidated or either personally threatens to hurt the woman or someone they cared for; *Sexual violence*: verbally forced to have sexual intercourse, physically forced to have sexual intercourse, accepting sexual intercourse because of fear of partners reaction, or forced to do a sexual act which the women found degrading or humiliating.

**Birth outcomes**: Preterm birth and Low birth weight.

#### Co-variates

Information on socio-demographic characteristics (age, education level, and occupation), lifestyle factors (smoking and alcohol consumption), previous history of adverse pregnancy outcomes, and chronic morbidities (diabetes mellitus, hypertension) were collected at inclusion.

#### Biological markers

The women’s blood pressure, haemoglobin level, weight and height were measured at inclusion and BMI was calculated. Information of the women’s HIV test status was obtained from the women’s antenatal card.

### Statistical analysis

Data were double entered in EpiData and exported to Stata version 10 for statistical analyses (S1 File, S2 File). Experience of any physical, sexual or emotional violence was considered if a woman reported being exposed to at least one of the acts of violence exerted by her partner during the present pregnancy. To estimate the association between maternal exposure to physical violence and risk of PTB and LBW, logistic regression analyses were performed and odds ratios (OR) with 95% confidence intervals (CI) were calculated. Firstly, crude ORs were calculated and secondly possible confounders were controlled for in multivariate logistic regression analyses. In the first regression model additional adjustment for emotional and sexual violence was performed, in the second regression model additional adjustment for the effect of maternal age, education, occupation, previous adverse pregnancy outcome, BMI, haemoglobin status was performed. CIs were calculated at the 95% level. To identify effect modification, stratified analyses of the association between physical violence and PTB and LBW, respectively, were performed on the basis of age, education, occupation, previous adverse pregnancy outcomes, BMI and haemoglobin level. No significant differences were found across the different strata.

### Ethical consideration

The women were informed in detail about the study and written informed consent was obtained. The study proposal was submitted to and approved by the Ethical Committee of Hanoi Medical University. We followed the WHO ethical and safety recommendations for researching domestic violence against women. If the pregnant women were found to be exposed to IPV, she was firstly counselled by the research team about supportive possibilities and if she accepted, referral to professional supportive agencies was arranged.

## Results

In all 1276 out of 1337 women, equal to a follow up rate of 96%, were interviewed after delivery. The women who were lost for follow up did not differ from the study population in terms of socioeconomic characteristics; their mean age was 26.7 years, 80% were educated above secondary school and 32% were workers.

Data from the 1276 pregnant women were analysed and the women’s baseline characteristics are listed in Table 1. Seventy-nine women (6.2%) had a PTB and sixty-two women (4.9%) gave birth to a LBW infant. The prevalence of any violence during pregnancy was 35.4%. The prevalence of previous PTB, LBW, miscarriages and still birth were 2.7%; 2.4%; 13.2% and 9.7%, respectively. The most common form of violence was emotional violence; this was reported by 32.5% of the women. Ten percent of the women had experienced sexual violence and 3.5% reported physical violence during their pregnancy. One of four women had anemia (25%) and 17.9% of the women had a BMI < 18.5. Smoking and alcohol consumption were not common, only 10 women (0.8%) stated they had been smoking and 80 women (6.3%) that they had been drinking alcohol during their present pregnancy.

**Table 1:** Socio-demographic background and prevalence of exposure to at least one type of violence during pregnancy.

	Any Violence (Emotional or physical or sexual violence)		
	Number of women who were not exposed to IPV (%)	Number of women who were exposed to IPV (%)	Number of women (% of total)
**Age (years)**			
- 17–19	41 (5)	34 (7.5)	75 (5.9)
- 20–29	578 (70.1)	316 (69.9)	894 (70.1)
- 30–34	143 (17.4)	81 (17.9)	224 (17.5)
- ≥ 35	62 (7.5)	21 (4.7)	83 (6.5)
**Education**			
- Less than high school	149 (18.1)	102 (22.6)	251 (19.7)
- High school	297 (36)	172 (38.0)	469 (36.7)
- Above high school	378 (45.9)	178 (39.4)	556 (43.6)
**Occupation**			
- Employee	305 (37)	107 (23.7)	412 (32.3)
- Worker	209 (25.4)	137 (30.3)	346 (27.1)
- Farmer	95 (11.5)	73 (16.1)	168 (13.2)
- Other	215 (26.1)	135 (29.9)	350 (27.4)
**Anaemia**			
- No (Hb ≥ 110 g/ml)	627 (76.1)	330 (73.0)	957 (75)
- Yes (Hb < 110 g/ml)	197 (23.9)	122 (27)	319 (25)
**BMI**			
- BMI < 18.5	154 (18.7)	74 (16.4)	228 (17.9)
- 18.5–24.9	625 (75.8)	355 (78.5)	680 (76.8)
- BMI≥ 25	45 (5.5)	23 (5.1)	68 (5.3)
**Smoking during pregnacy**			
- No	818 (99.3)	448 (99.1)	1266 (99.2)
- Yes	6 (0.7)	4 (0.9)	10 (0.8)
**Drinking Alcohol/Beer during pregnacy**			
- No	772 (93.7)	424 (93.8)	1196 (93.7)
- Yes	52 (6.3)	28 (6.2)	80 (6.3)
**Economic status**			
- Quintile 1	265 (32.2)	182 (40.3)	447 (35)
- Quintile 2	427 (51.8)	213 (47.1)	640 (50.2)
- Quintile 3	132 (16)	57 (12.6)	189 (14.8)
**Previous preterm birth**			
- No	804 (97.6)	438 (96.9)	1242 (97.3)
- Yes	20 (2.4)	14 (3.1)	34 (2.7)
**Previous low birth weight**			
- No	811 (98.4)	434 (96)	1245 (97.6)
- Yes	13 (1.6)	18 (4)	31 (2.4)
**Miscarriages**			
- No	719 (87.3)	389 (86.1)	1108 (86.8)
- Yes	105 (12.7)	63 (13.9)	168 (13.2)
**Still birth**			
- No	741 (89.9)	411 (90.9)	1152 (90.3)
- Yes	83 (10.1)	41 (9.1)	124 (9.7)
**Low birth weight**			
- No	789 (95.8)	425 (94)	1214 (95.1)
- Yes	35 (4.2)	27 (6)	62 (4.9)
**Preterm birth**			
- No	773 (93.8)	424 (93.8)	1197 (93.8)
- Yes	51 (6.2)	28 (6.2)	79 (6.2)
**Total**	**824 (100)**	**452 (100)**	**1276 (100)**

We analysed how emotional violence, sexual and physical violence were associated with PTB and LBW (Table 2). Emotional or sexual violence was not found to be significantly associated with PTB or LBW, whereas a significant association was found between physical violence and PTB or LBW. In all 45 women had been exposed to physical violence, the vast majority (n = 44) in combination with either emotional and/or sexual violence. An increasing risk of PTB or LBW was found among women who were exposed to different types of violence at the same time. More specifically, women who were exposed to both physical and emotional violence had increased ORs of 6.6 (95% CI: 2.6–16.5) and 4.4 (95% CI: 1.8–10.8) of experiencing LBW or PTB, respectively. If the women were exposed to physical, emotional as well as sexual violence at the same, the OR for LBW was further increased to 10 (95% CI: 2.9–34.7) whereas the OR for PTB did not change much.

**Table 2:** Association between different combinations of violence during pregnancy and birth outcome.

	LBW No n = 1214 (%)	LBW Yes n = 62 (%)	OR (95%CI)	PTB No n = 1197 (%)	PTB Yes n = 79 (%)	OR (95%CI)
**No violence**	789 (65)	35 (56.5)	1	773 (64.6)	51 (64.5)	1
**Only emotional violence**	284 (23.4)	10 (16.1)	0.8 (0.4–1.6)	285 (23.8)	9 (11.4)	0.5 (0.2–1)
**Only sexual violence**	35 (2.9)	1 (1.6)	0.6 (0.1–4.8)	33 (2.8)	3 (3.8)	1.4 (0.4–4.6)
**Only physical violence**	1 (0.1)	0 (0)	-	1 (0.1)	0 (0)	-
**Emotional and sexual violence**	72 (5.9)	5 (8.1)	1.6 (0.6–4.1)	71 (5.9)	6 (7.6)	1.3 (0.5–3.1)
**Physical and emotional violence**	24 (2)	7 (11.3)	6.6 (2.6–16.5)	24 (2)	7 (8.9)	4.4 (1.8–10.8)
**Physical and sexual and emotional violence**	9 (0.7)	4 (6.4)	10.0 (2.9–34.0)	10 (0.8)	3 (3.8)	4.5 (1.2–17.1)

The association between PTB and possible risk factors are presented in Table 3. Women who were exposed to physical violence had five times increased OR of PTB (AOR = 5.5, 95% CI: 2.1–14.1) compared to women who were not exposed. We also found an association between previous adverse pregnancy outcomes and PTB, where women had an increased OR of PTB in relation to their present pregnancy if they had experienced a previous PTB (AOR = 3.8; 95%CI: 1.3–11.2).

**Table 3:** The association between IPV during pregnancy and preterm birth.

	Number of women n = 1276 (%)	Number of women who had PTB n = 79 (%)	Crude OR (95%CI)	Model 1* AOR (95%CI)	Model 2** AOR (95%CI)
**Previous preterm**					
- No	1242 (97.3)	73 (92.4)	1	1	1
- Yes	34 (2.7)	6 (7.6)	3.4 (1.4–8.6)	3.6 (1.4–8.9)	3.8 (1.3–11.2)
**Previous low birth weight**					
- No	1245 (97.6)	74 (93.7)	1	1	-
- Yes	31 (2.4)	5 (6.3)	3.0 (1.1–8.2)	3.2 (1.2–8.6)	
**Miscarriages**					
- No	1108 (86.8)	69 (87.3)	1	1	-
- Yes	168 (13.2)	10 (12.7)	0.95 (0.5–1.9)	0.9 (0.5–1.9)	
**Still birth**					
- No	1152 (90.3)	70 (88.6)	1	1	-
- Yes	124 (9.7)	9 (11.4)	1.2 (0.6–2.5)	1.2 (0.6–2.5)	
**Emotional violence**					
- No	861 (67.5)	54 (68.4)	1	-	-
- Yes	415 (32.5)	25 (31.7)	1.0 (0.6–1.6)		
**Physical violence**					
- No	1231 (96.5)	69 (87.3)	1	1	1
- Yes	45 (3.5)	10 (12.7)	4.8 (2.3–10.2)	6.3 (2.7–15)	5.5 (2.1–14.1)
**Sexual violence**					
- No	1150 (90.1)	67 (84.8)	1	-	-
- Yes	126 (9.9)	12 (15.2)	1.7 (0.9–3.2)		

* Model 1: Adjusted for emotional violence, sexual violence

** Model 2: Adjusted for emotional violence, sexual violence, previous low birth weight, miscarriages, stillbirth, women’s age, education, occupation, BMI, anemia status

Table 4 shows the crude and adjusted OR’s of LBW according to previous adverse pregnancy outcome and exposure to IPV during pregnancy. We found a strong relationship between exposure to physical violence during pregnancy and LBW. Women who were exposed to physical violence during pregnancy had nearly six times increased OR for giving birth to a LBW infant (AOR = 5.7; 95% CI: 2.2–14.9) as women not expose.

**Table 4:** The association between IPV during pregnancy and low birth weight.

	Number of women n = 1276 (%)	Number of women who had LBW n = 62 (%)	Crude OR (95%CI)	Model 1* AOR (95%CI)	Model 2** AOR (95%CI)
**Previous preterm**					
- No	1242 (97.3)	58 (93.6)	1	1	-
- Yes	34 (2.7)	4 (6.5)	2.7 (0.9–8)	2.8 (0.9–8.1)	
**Previous low birth weight**					
- No	1245 (97.6)	57 (91.9)	1	1	1
- Yes	31 (2.4)	5 (8.1)	4.0 (1.5–10.9)	3.7 (1.3–10.1)	2.4 (0.7–8.1)
**Miscarriages**					
- No	1108 (86.8)	56 (90.3)	1	1	-
- Yes	168 (13.2)	6 (9.7)	0.7 (0.3–1.6)	0.7 (0.3–1.6)	
**Still birth**					
- No	1152 (90.3)	56 (90.3)	1	1	-
- Yes	124 (9.7)	6 (9.7)	1.0 (0.4–2.4)	1.0 (0.4–2.4)	
**Emotional violence**					
- No	861 (67.5)	36 (58.1)	1	-	-
- Yes	415 (32.5)	26 (41.9)	1.5 (0.9–2.6)		
**Physical violence**					
- No	1231 (96.5)	51 (82.3)	1	1	1
- Yes	45 (3.5)	11 (17.7)	7.5 (3.5–15.8)	7.3 (3.2–17.1)	5.7 (2.2–14.9)
**Sexual violence**					
- No	1150 (90.1)	52 (83.9)	1	-	-
- Yes	126 (9.9)	10 (16.1)	1.8 (0.9–3.7)		

* Model 1: Adjusted for emotional violence, sexual violence

** Model 2: Adjusted for emotional violence, sexual violence, previous preterm, miscarriages, stillbirth, women’s age, education, occupation, BMI, anemia status

We performed stratified analyses based on history of previous adverse pregnancy outcome (PTB, LBW, miscarriages, still birth) and examined the association between IPV and LBW or PTB among women with a history of previous adverse pregnancy outcomes and among women without such a history. The results are summarized in Table 5. Exposure to IPV, among women who previously had experienced an adverse pregnancy outcome, was associated with an increased OR of PTB of 13.3 (95% CI: 2.5–69.9) and an increased OR of LBW of 9.4 (95% CI: 2.0–44.3). IPV exposure among women, who had not previously experienced an adverse pregnancy outcome, was not increased to the same extent with corresponding ORs of 5.3 (95% CI: 1.8–15.2) and 7.2 (95% CI: 2.6–20.4), for PTB and LBW respectively.

**Table 5:** The association between physical violence and PTB or LBW among women without previous adverse obstetric history and women with previous adverse obstetric history.

	Number of women (%)	Preterm Birth			Low Birth Weight		
		Number of women who had PTB (%)	Crude OR (95%CI)	AOR* (95%CI)	Number of women who had LBW (%)	Cure OR (95%CI)	AOR* (95%CI)
**WITH previous adverse obstetric history (n = 298)**							
**Physical violence**							
- No	285 (95.6)	18 (81.8)	1	1	11 (73.3)	1	1
- Yes	13 (4.4)	4 (18.2)	6.6 (1.3–26.3)	13.3 (2.5–69.9)	4 (26.7)	11.1 (2.1–47.3)	9.4 (2.0–44.3)
**WITHOUT previous adverse obstetric history (n = 978)**							
**Physical violence**							
- No	946 (96.7)	51 (89.5)	1	1	40 (85.1)	1	1
- Yes	32 (3.3)	6 (10.5)	4.0 (1.3–10.6)	5.3 (1.8–15.2)	7 (14.9)	6.3 (2.2–16.2)	7.2 (2.6–20.4)

* Adjusted for emotional violence, sexual violence.

## Discussion

A strong relationship between exposure to physical violence during pregnancy and PTB or LBW was found. Our research further showed that IPV is common among pregnant women in Vietnam.

More than one-third of the women had been exposed to IPV during pregnancy; 32.5% reported exposure to emotional, 3.5% to physical and 10% to sexual violence. In comparison, a recent meta-analysis focusing on IPV during pregnancy, reported the prevalence of emotional violence to be 28.4%, physical violence to be 13.8%, and sexual violence to be 8.0% [16]. Considerably higher rates of IPV during pregnancy has been reported in a study from Thailand where 54% of the women had been exposed to emotional violence, 27% to physical violence and 19% to sexual violence [17]. When focusing on exposure to physical violence during pregnancy, another meta-analysis based on survey data form 19 countries has reported prevalence rates from 2.0% to 13.5% with rates being higher in African and Latin American countries relative to European and Asian countries [2]. The variations in the reported prevalence rates of pregnancy IPV likely reflect regional variations and differences in study methodologies and consequently it is difficult to reach any comparative conclusions.

The prevalence of PTB and LBW in our study was 6.2% and 4.9%. A finding which is lower than the prevalence in developed countries where estimates of 9.6% PTB and 15.5% LBW have been reported [18, 19]. Niemi and et al using last menstrual period to measure PTB and LBW estimated 19.8% PTB and 9% LBW [20]. However, the author also explained that the high prevalence of PTB and LBM might reflect that women have difficulties in remembering the exact date of the last menstrual period, which may be subject to recall error with a tendency to overstate the duration of pregnancy. Studies relying on last menstrual period may therefere be associated with falsely high prevalence rates of PTB and LBW [21, 22]. Since our study relied on ultrasound based gestational age determination, we believe the findings are valid. They are further well in line with the national reported incidence rates of PTB (5.2%) and LBW (7.1%) [13]. The slightly higher rate found in our study can be explained by the fact that Dong Anh is a district of Hanoi–the capital in Vietnam–with households of better economic conditions and thus better nutrition and health status than the average level nationwide.

We discovered a strong relationship between exposure to IPV and occurrence of PTB or LBW. Other studies have similarly found an association between IPV exposure and PTB or LBW. A prospective cohort study from United States, documented that IPV during pregnancy was associated with a 4-fold increased risk for having a LBW infant [23]. Further, a literature review from 2014 in Latin American and Caribbean reported that IPV during pregnancy was associated with an increased risk of PTB, LBW, neonatal complications and stillbirth [24]. Two other cohort studies from Brazil and Iran have reported similar results, although with varying strength of the association. Hence in Brazil, exposure to physical violence during pregnancy was found to be associated with a 2.2-fold increased risk of LBW [12]. Whereas the Iranian study showed that IPV during pregnancy was associated with a 1.9 fold increased risk of PTB and a 2.9 fold increased risk of LBW [25]. Likewise, a recent study from Egypt conducted among 1,857 pregnant women suggest that IPV exposure is associated with a significantly higher incidence of adverse pregnancy outcomes (miscarriage, preterm labour, and premature rupture of membrane) as well as adverse fetal and neonatal outcomes (fetal distress, fetal death, and low birth weight) [26]. Finally, a randomized controlled trial conducted among African-Americans from 2001 to 2003 concluded that IPV was associated with increased risk of PTB [15].

We also found that the risk of PTB or LBW increased when the pregnant women were exposed to more than one type of violence. This is in agreement with a case-control study from Peru where isolated emotional violence during pregnancy was found to be associated with a 1.6-fold increased risk of PTB. If the woman was exposed to both emotional and physical abuse during pregnancy the risk increased to 4.7 [27].

Exposure to different type of violence may affect the woman both physically and mentally and can lead to PTB or LBW through direct or indirect mechanisms. Hence, available evidence suggests that physical IPV may lead to traumas causing premature rupture of membranes or cause abruption of the placenta and subsequently PTB and LBW [28]. IPV can also lead to adverse pregnancy outcome through a number of indirect mechanisms. It has been put forward that IPV may lead to chronic psychological stress and elevated levels of stress hormones such as e.g. cortisol that may hamper the body’s immune function and that may be associated with increased risk of preterm labour [29]. Further, exposure to emotional, physical and sexual violence may in addition have an impact on the pregnancy through alterations in the woman’s psychological wellbeing and lifestyle habits. Hence, impaired psychological wellbeing may lead to hypertension or preeclampsia, which may be associated with poor foetal weight gain and preterm delivery. Moreover, violence exposure may lead to alterations in lifestyle habits as e.g. increased smoking, alcohol or drug abuse which may also affect the health of the women as well as the foetus [30, 31].

To our knowledge this is the first prospective cohort study about IPV during pregnancy in Vietnam. In the interpretation of the results there are some important limitations which should be considered. Firstly, we relied on a cohort design which imposes a risk of differential loss to follow up. To address this problem, we involved community collaborators as research assistants, an approach which helped us identifying the women postpartum and thus helped ensuring a high follow up rate of 96%. Due to this high follow up rate, we believe that the risk of selection bias is negligible. Secondly, accurate estimation of gestational age is a challenge in many low and middle income countries and incorrect estimation is associated with risk of misclassification of PTB and LBW. This problem was addressed through the involvement of medical doctors who were qualified in performing obstetrical ultrasound scanning. Based on the scanning, which were performed before 24 weeks of gestation, the gestational age at birth was determined. Thirdly, IPV is a sensitive topic and women exposed to IPV might have been reluctant to share their experiences for fear of their husbands and their communities’ reactions. To address this problem, all research assistants were middle-aged women who were trained carefully in utilizing an empathetic approach while interviewing. Hence, the interviewer would make a safe and comfortable environment for women usually a private room at a community health centre or hospital. To create an atmosphere of confidentiality, the interviews were conducted as a conversation where the interviewers were encouraged to share their own living experiences with the women when considered relevant. All information was kept secret and the study was named “life experience” rather than “intimate partner violence” throughout the data collection period to avoid revealing that the study focused on IPV. Finally, information on possible confounders was obtained at inclusion and multiple logistic regressions were used to control for their impact on the association between IPV and PTB and LBW, however, although we adjusted for most recognized confounders, the possibility of residual confounding (e.g. changes in nutritional status and changes in maternal weight) cannot be excluded. [32, 33].

## Conclusion

Our study showed that violence is common during pregnancy and that physical violence during pregnancy associated with increased risk of PTB and LBW National programs targeting violence against women should therefore emphasize that violence exercised during pregnancy has very serious health implications.

## Supporting Information

S1 File Complete dataset. (XLSX)

S2 File Label for the variables in the dataset. (XLSX)
